# Optimization Course of Titanium Nitride Nanofiller Loading in High-Density Polyethylene: Interpretation of Reinforcement Effects and Performance in Material Extrusion 3D Printing

**DOI:** 10.3390/polym16121702

**Published:** 2024-06-14

**Authors:** Markos Petousis, Dimitris Sagris, Vassilis Papadakis, Amalia Moutsopoulou, Apostolos Argyros, Constantine David, John Valsamos, Mariza Spiridaki, Nikolaos Michailidis, Nectarios Vidakis

**Affiliations:** 1 Department of Mechanical Engineering, Hellenic Mediterranean University, 71410 Heraklion, Greece; markospetousis@hmu.gr (M.P.); amalia@hmu.gr (A.M.); valsamos@hmu.gr (J.V.); mspyridaki@hmu.gr (M.S.); 2 Department of Mechanical Engineering, Serres Campus, International Hellenic University, 62124 Serres, Greece; dsagris@ihu.gr (D.S.); david@ihu.gr (C.D.); 3 Institute of Electronic Structure and Laser of the Foundation for Research and Technology-Hellas (IESL-FORTH)–Hellas, N. Plastira 100 m, 70013 Heraklion, Greece; v.papadakis@uniwa.gr; 4 Department of Industrial Design and Production Engineering, University of West Attica, 12243 Athens, Greece; 5 Physical Metallurgy Laboratory, Mechanical Engineering Department, School of Engineering, Aristotle University of Thessaloniki, 54124 Thessaloniki, Greece; aargyros@auth.gr (A.A.); nmichail@auth.gr (N.M.); 6 Centre for Research & Development of Advanced Materials (CERDAM), Centre for Interdisciplinary Research and Innovation, Balkan Centre, Building B’, 10th km Thessaloniki-Thermi Road, 57001 Thessaloniki, Greece

**Keywords:** high-density polyethylene (HDPE), titanium nitride (TiN), nanocomposites, material extrusion (MEX), three-dimensional printing (3D-P), mechanical testing

## Abstract

In this study, titanium nitride (TiN) was selected as an additive to a high-density polyethylene (HDPE) matrix material, and four different nanocomposites were created with TiN loadings of 2.0–8.0 wt. % and a 2 wt. % increase step between them. The mixtures were made, followed by the fabrication of the respective filaments (through a thermomechanical extrusion process) and 3D-printed specimens (using the material extrusion (MEX) technique). The manufactured specimens were subjected to mechanical, thermal, rheological, structural, and morphological testing. Their results were compared with those obtained after conducting the same assessments on unfilled HDPE samples, which were used as the control samples. The mechanical response of the samples improved when correlated with that of the unfilled HDPE. The tensile strength improved by 24.3%, and the flexural strength improved by 26.5% (composite with 6.0 wt. % TiN content). The dimensional deviation and porosity of the samples were assessed with micro-computed tomography and indicated great results for porosity improvement, achieved with 6.0 wt. % TiN content in the composite. TiN has proven to be an effective filler for HDPE polymers, enabling the manufacture of parts with improved mechanical properties and quality.

## 1. Introduction

For more than 20 years, researchers and developers have focused on advancing additive manufacturing (AM) technology, which is constantly being increasingly utilized [1,2] and becoming popular in the industrial sector [3,4,5,6]. It is based on manufacturing parts through three-dimensional (3D) printing [7] by repeatedly adding layers of the chosen materials. These parts were designed to have flexibility, complexity, larger dimensions, and lower prices [8,9,10,11,12,13,14,15]. There are various applications in various fields, such as aerospace [16,17], electronics [18,19], automotive [20], biomedical, energy, architecture, arts, food design, and construction [21,22]. It can be useful in cases such as the low-quantity production of single industrial products, customized medical implants, cultural or creative displays, and other personalized daily necessities [23,24,25,26]. In addition, the lightweight parts are used in many industries, such as aerospace [27,28,29,30,31] and fast prototyping in the automotive industry [32,33]. AM enables the production of parts with complex designs, low manufacturing costs, reduced material wastage, and multifunctionality [34]. The existing AM processes can utilize materials that vary from resins to ceramics or metals, depending on the current process [35,36,37,38], as well as polymers, such as thermoplastics, thermosets, polymer blends, and polymer-based composites [39].

Thermoplastics are characterized by their simple molecular structures and chemically independent macromolecules [40,41]. After melting or softening during heating, they can be shaped, formed, welded, and solidified by cooling [42]. Reprocessing and recycling are possible because they can undergo many heating and cooling transitions, while avoiding damage [43]. It is common for thermoplastics to be filled with additives to enhance their thermal, chemical, and ultraviolet resistance [44,45]. Some of the most known categories of thermoplastics are polypropylene (PP), Polyvinyl Chloride (PVC), Polystyrene (PS), Polyethylene (PE), polyamide (PA), Acrylonitrile-Butadiene-Styrene (ABS), Polyether ether Ketone (PEEK), Polycarbonate (PC), etc. [46].

Polyethylene encompasses a range of types, including LDPE and MDPE, which are commonly used for flexible applications such as bags and packaging. Its density ranges between 0.88 and 0.96 g/cm^3^. Besides Polyethylene (PE) and polyethylene terephthalate (PET), which are the most commonly utilized plastics [47], high-density polyethylene (HDPE), with the chemical formula structure (C_2_H_4_)n linear and a density of 0.96 g/cm^3^, is the third most commonly used plastic in the field of construction [48]. High-density polyethylene is characterized by its strength and rigidity and is well suited for robust applications, such as pipes, containers, and construction materials. HDPE has a linear and opaque structure. It also has a higher cost than PE.

HDPE belongs to the category of thermoplastics that are utilized extensively in 3D printing because it is resistant to both high and low temperatures, chemically stable and mechanically strong, and has electrical isolation and barrier qualities [49]. It can be used in various applications, such as automotive parts, packaging, agriculture, and other dairy products [50,51]. HDPE is often chosen as the matrix material in polymeric composites due to its qualities, such as its flexibility and deformability [52]. It has also been used as one of the materials employed for the creation of composites in previous studies in the existing literature [53,54,55,56,57,58,59,60,61,62]. The fillers that have been combined with HDPE in the previous studies for injection molding and 3D printing applications include carbon [63], calcium carbonate [64], graphite nanofibers [65], fly ash cenospheres [66,67], glass micro balloons [68,69,70], biochar [44], and copper for antibacterial and defense applications [45].

Titanium nitride (TiN) belongs to the category of ceramic additives that are known to enhance the performance of materials [71]. Therefore, they are used in applications where the materials are subjected to extreme conditions, such as cutting tools [72]. TiN can be utilized in many applications, especially as a coating [73] to increase the wear resistance of surfaces [74,75,76], as well as for medical reasons (implants) [77,78,79], energy applications [80], the prevention of electromagnetic interference, and optical applications [81,82,83,84,85,86]. Material extrusion (MEX) 3D printing has been proven to be an efficient reinforcing agent for different matrices, such as Acrylonitrile Butadiene Styrene (ABS) [71], polyamide 12 [87], polypropylene (PP) [88], Polylactic Acid (PLA) [89], and Polycarbonate (PC) [90]. HDPE/TiN composites have been presented in the literature before, but not in 3D printing, and the existing research focuses on wear resistance increase due to the introduction of a TiN filler in the composites [91,92].

In this study, HDPE was combined with TiN fillers of various concentrations to evaluate the effect of their addition on the mechanical performance and overall response of the matrix. The composites were used to create filaments, which were used for the fabrication of specimens appropriate for conducting flexural, impact, and tensile tests. The samples were subjected to scanning electron microscopy (SEM), differential scanning calorimetry (DSC), thermogravimetric analysis (TGA), and Raman spectroscopy. The aim of this study was to investigate the mechanical performance and reinforcing ability of HDPE caused by the addition of TiN. To the best of our knowledge, no investigations have been conducted on the mechanical, thermal, or structural responses of HDPE/TiN composites in MEX 3D printing. The current study aims to achieve the following:

- Assess the ability of TiN to enhance the performance of HDPE.
- Create HDPE/TiN composites suitable for use as materials for a wide variety of applications to meet the demands.
- Characterize the composites to verify their suitability for real-life applications.

The effects of TiN nanoparticles on the 3D printed parts’ dimensional accuracy and porosity are metrics related to their build quality.

## 2. Materials and Methods

**Figure 1:**
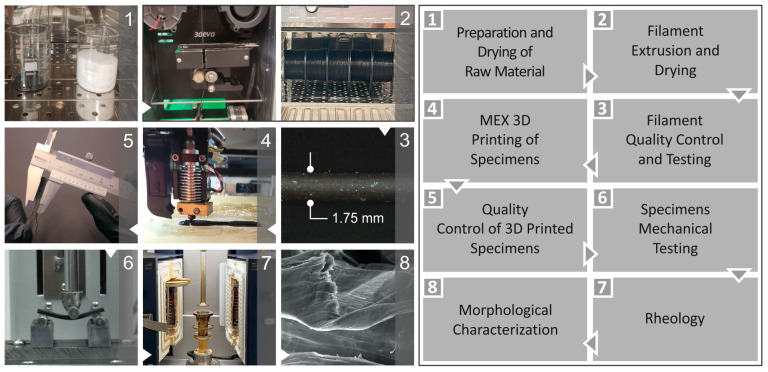
The experimental procedure’s steps are (**1**) raw material preparation and drying**,** (**2**) filament extrusion, and drying, (**3**) the quality inspection and testing of filaments, (**4**) the material extrusion 3D-P and (**5**) mechanical testing of specimens, (**6**) the rheologic evaluation of samples**,** (**7**) dielectric and conductivity characterization, and (**8**) morphological characterization.

The process is illustrated in Figure 1. This comprises the preparation of raw materials, fabrication through the thermomechanical extrusion method of filaments (which were then dried prior to their use for the 3D printing of samples), and the assessment of their quality, followed by their testing, which is illustrated in this work. Subsequently, for the production of samples, the 3D printing (3D-P) technique of material extrusion (MEX) was utilized, followed by mechanical testing, and rheological, dielectric, and morphological characterizations were performed.

### 2.1. Materials

Industrial-grade high-density polyethylene (HDPE) powder was used as the matrix in this study. According to the data provided by the supplier, the melt mass flow rate (MFR) at 190 °C and 2.16 kg was 7.5 g/10 min, the material density was 0.960 g/cm^3^, and the Vicat softening temperature as 127 °C. The additive used was titanium nitride (TiN), which was provided by Nanographi (Ankara, Turkey). Its properties were 99.2% purity, a particle size of 20 nm, a cubic shape, and 5.3 g/cm^3^ density.

### 2.2. Scanning Electron Microscopy (SEM) and Energy-Dispersive Spectroscopy (EDS) Analysis

**Figure 2:**
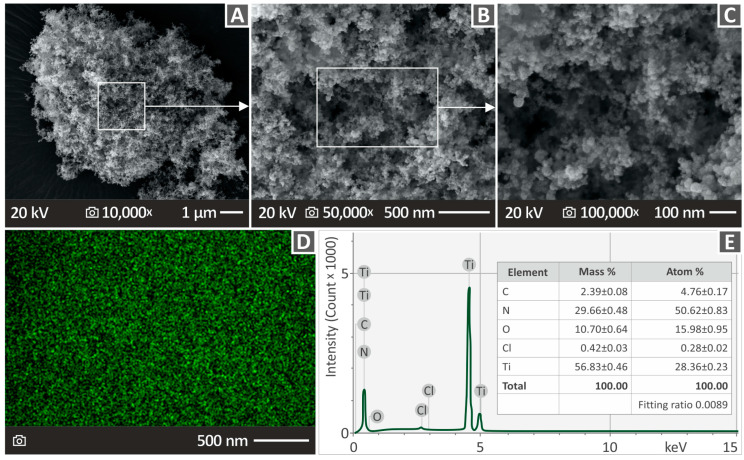
(**A**–**C**) SEM pictures of TiN material magnified at 10,000×, 50,000×, and 100,000×. (**D**) Picture from EDS mapping (for the element of Ti) of the TiN. (**E**) Chemical composition of TiN provided by EDS analysis.

Scanning electron microscopy (SEM) and energy-dispersive spectroscopy (EDS) (which were implemented using the same apparatus) were used to examine the structural, morphological, and chemical compositions. The apparatus used for both SEM and EDS was a JEOL (Tokyo, Japan) JSM-IT700HR field-emission SEM. SEM images were obtained from the fracture and lateral surfaces of the samples as well as the TiN material, and their chemical compositions were assessed using EDS. Figure 2 shows the SEM and EDS results for TiN. Figure 2A–C shows the SEM images of TiN magnified at 10,000×, 50,000×, and 100,000×. Figure 2D shows the EDS MAP of TiN (for titanium, green dots), while Figure 2E shows the EDS analysis of the chemical composition of TiN. As expected, high levels of Ti were detected. It should be noted that the presence of oxygen is related to the process followed to acquire images of TiN nanoparticles. The nanoparticles were placed on carbon tape adhered to laboratory glass. Therefore, the presence of oxygen can be attributed to the presence of carbon tape in the observation area. Additionally, the increased number of N atoms can be attributed to carbon tape used, as well as to the TiN nanoparticles, which have N in their composition, according to the elemental analysis of the manufacturer, with a ratio of approximately ½ to the number of Ti atoms.

### 2.3. Composite Preparation, Filaments, and 3D Specimen Fabrication

The desired quantities of the matrix material and additive material were prepared and used to create four different composites, namely HDPE/TiN 2.0–8.0 wt. % (with a 2.0 step). Nanocomposites were prepared using a thermomechanical extrusion process. The raw materials were thoroughly mixed using a blender (4000 rpm for 30 min at room temperature) and placed in an oven to dry overnight (~8 h) at approximately 80 °C. No other additives were used to evaluate the effects of additives in the matrix material. Subsequently, they were inserted into a 3D Evo model Composer 450 extruder (3devo B.V., Utrecht, The Netherlands) to fabricate filaments corresponding to each mixture. This is a specifically designed single-screw extruder for material and filler mixing, as claimed by its manufacturer, owing to the special design of its screw. The extruder was accompanied by a filament diameter sensor operating in a closed-loop scheme to ensure an acceptable filament diameter during its production. The necessary micro-adjustments were made to the extrusion speed in order for the diameter filament to be maintained between 1.65 mm and 1.85 mm and be 3D-P-suitable. Furthermore, the filament was inspected for quality before its utilization in 3D-P.

The filler percentage was chosen after gradually increasing loading, testing the samples, and considering the results provided by the tests. In particular, when the mechanical properties started to deteriorate, filler loading did not increase further. This was because it was anticipated that additional loading would not improve the mechanical properties owing to the saturation of the TiN nanoparticles in the matrix. The parameters for extrusion and 3D printing were set based on information provided in the literature [93].

**Figure 3:**
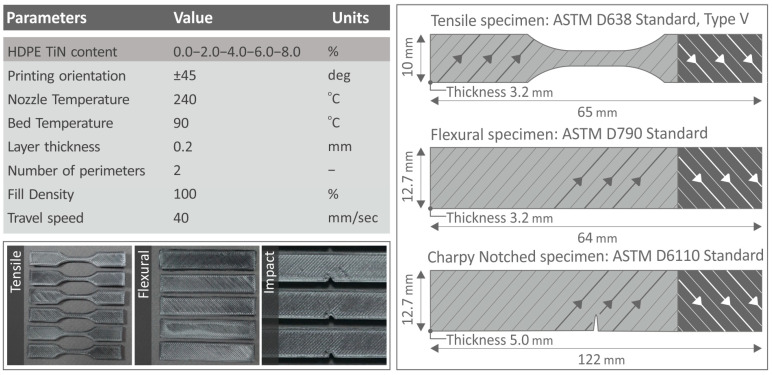
(**Top left side**) a board listing the printing settings of the 3D parts, (**bottom left side**) pictures of the manufactured test samples, (**right side**) and their geometry and infill pattern of the 3D printing structure (strands orientation shifts 90 degrees between successive layers), along with dimensions [94,95,96].

HDPE/TiN specimens were manufactured using an MEX 3D printer model named Funmat HT 3D by Intamsys Company (Intamsys Technology Co., Ltd., Shanghai, China). The Autodesk^®^ Fusion 360™ v.2 (Autodesk^®^, Inc., San Francisco, CA, USA) software platform was used to create a 3D-P specimen design that was suitable for mechanical testing. The 3D-P settings utilized are shown in Figure 3.

### 2.4. Mechanical Tests

The mechanical testing implemented within the context of this research was as follows:

- Tensile testing was performed according to the ASTM D638-02a international standard. The samples were V-type tensile specimens having a height of 3.2 mm, and the utilized device was a tension/flexure test Imada MX2 from Imada Inc. (Northbrook, IL, USA) in tensile mode, accompanied by standardized grips.
- Flexural testing was based on the ASTM D790-10 international standard. The type of test was a three-point bending test, and the distance between the supports was set at 52.0 mm. Testing was also carried out using the same device as that used for tensile testing, while being in the appropriate setup for flexural testing.
- Charpy impact testing followed the instructions of the ASTM D6110-04 international standard. The apparatus used was a model MT 220 (Terco, Kungens Kurva, Sweden), compatible with the Charpy protocol for impact testing.
- Vickers microhardness measurements were performed according to ASTM E384-17 [97]. They were performed using the Innova Test model name 300 from Innovatest (Europe BV, Maastricht, The Netherlands). Prior to microhardness measurements, the surfaces of the specimens were meticulously polished. The samples were subjected to a 100 g force (gF) for a time period of 10 s to create the indentation.

Figure 3 shows a board listing the printing parameter set for the construction of 3D-P samples. Figure 3 illustrates the geometry of the test samples for each mechanical test.

### 2.5. Raman Spectra

Raman spectra were obtained under controlled laboratory conditions using a confocal LabRAM HR Raman spectrometer (HORIBA Scientific, Kyoto, Japan). The spectrometer’s primary laser line, with a power of 90 mW at 532 nm, was used for excitation. To decrease the laser power irradiating the sample, a 5% Neutral-Density filter was positioned in the optical path. A 50× objective lens from LMPlanFL N (Olympus, Tokyo, Japan) with a Numerical Aperture (NA) of 0.5× and a working distance of 10.6 mm was employed for imaging and excitation. Data acquisition was performed using a microscope with the specified settings.

- The acquired spectra ranged between 50 and 3900 cm^−1^.
- The spectrometer was equipped with a grating of 600 grooves/mm, providing a spectral resolution of 2 cm⁻¹.
- The sample exposure time was 10 s at each measurement location.
- For statistical purposes, at each point, we acquired 5 accumulations.
- The imaging lateral resolution was 1.7 μm, whereas the axial resolution was 2 μm.
- The laser power measured at the surface of the sample was calculated to be 1.3 mW.

After each point measurement, the measured areas were microscopically inspected to detect any discoloration or degradation caused by laser irradiation. 

The raw Raman data were processed using LabSpec software v.6 (HORIBA, Kyoto, Japan). For each acquired spectrum, the same methodology was used as follows: (a) the cosmic rays were removed; (b) noise was removed from the signal with 5-point kernel; (c) data were cropped between 450 and 3050 cm^−1^; (d) the background was removed using an 11th-grade polynomial; (e) spectral data were recalibrated by the maximum peak; and (f) the resulting data were normalized using the maximum peak intensity.

### 2.6. Thermal Investigation

Thermogravimetric analysis (TGA) and differential scanning calorimetry (DSC) were performed to evaluate the thermal properties of pure HDPE. TGA was performed using a Diamond Perkin Elmer device (Waltham, Massachusetts, United States) in the temperature range 40–550 °C at a heating rate of 10 °C/min. DSC measurements were performed using a Discovery Series DSC-25 calorimeter (TA Instruments, New Castle, DE, USA) equipped with an RSC-90 Refrigerated Cooling System. Both the TGA and DSC experiments were conducted in an inert environment with high-purity N_2_ (nitrogen gas).

### 2.7. Viscosity and MFR

Rheometric measurements were performed using a DHR-20 Discovery Hybrid Rotational Rheometer (TA Instruments, New Castle, DE, USA). The melt flow rate (MFR) was determined according to the ASTM D1238-13 [98] guidelines. The apparatus included an Environmental Test Chamber equipped with a parallel plate setup for precise temperature control. To avoid excessive heating and decomposition, an acquisition time of 10 s was used for each measurement point. MFR and rotational rheometric tests were conducted to evaluate the material flow rates at specific temperatures and pressures.

### 2.8. Micro-Computed (μ-CT) Tomography

Two metrics related to the quality of the 3D-P parts, that is, the 3D printing structure’s porosity and dimensional accuracy, were measured using an accurate and reliable micro-computed tomography apparatus model Tomoscope HV Compact 225 kV Micro Focus produced by Werth Messtechnik GmbH (Giessen, Germany). The pixel sensor had a resolution of 1024 × 1024 pixels. The acquired data were processed using VG Studio MAX 2.2 (Werth Messtechnik GmbH, Giessen, Germany). Through this procedure, the effects of the addition of TiN particles to the HDPE matrix on these two quality metrics were evaluated. For dimensional accuracy, a 75 L setup was employed (the X-axis resolution was 72.58 μm, and the respective Y-axis resolution was 72.65 μm), while for porosity, a 16 L setup (the X-axis resolution was 15.46 μm, and the respective Y-axis resolution was 15.49 μm) was utilized. In both the dimensional accuracy and porosity measurements, 1600 sections were acquired to capture the structure of the parts accurately.

## 3. Results

### 3.1. Raman Results of HDPE/TiN Composites

**Figure 4:**
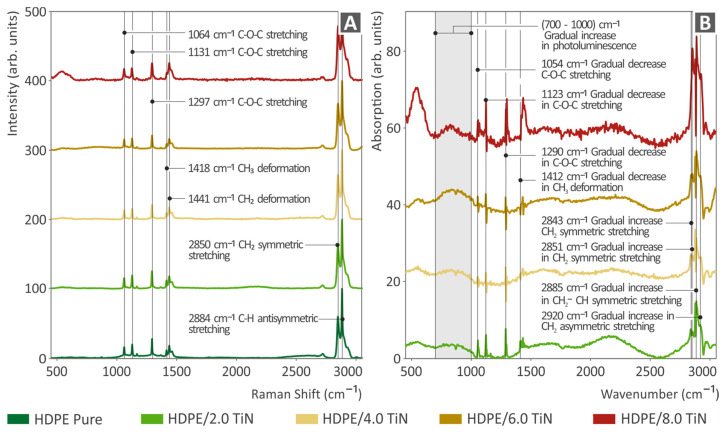
(**A**) Raman spectra of the unfilled HDPE and all THE composites prepared herein (HDPE/TiN, spectral curves are presented in stacked lines, with an offset of 0.25 difference between them). (**B**) Raman spectral variations between the unfilled HDPE and all the HDPE/TiN composites.

In Figure 4A, the Raman spectral profiles of the pure HDPE and HDPE/TiN mixtures are displayed. The Raman peaks of the pure HDPE sample were identified and compared with those in the literature [99,100,101,102,103], and details are provided in Table S1 in the Supplementary Materials.

As shown in Figure 4A, the Raman peaks identified in all the samples corresponded to unfilled HDPE. Specifically, C-O-C stretching was observed at 1064, 1131, and 1297 cm⁻¹. CH₃ and CH₂ deformations were observed at 1418 and 1441 cm⁻¹, respectively. Additionally, CH₂ symmetric stretching was observed at 2850 cm ⁻¹, and C-H antisymmetric stretching was observed at 2883 cm ⁻¹.

As shown in Figure 4B, there were clear differences from the pure HDPE. In particular, there was a gradual decrease in the Raman intensity at 1054, 1123, 1290, and 1412 cm^−1^ in the total amount of samples, showing a decrease in the concentration of the related bonds, as described in Table 1. Moreover, some Raman bands showed a gradual increase in intensity at 2843, 2851, 2885, and 2920 cm^−1^, indicating an increase in the concentration of the bonds, as described in Table 1. Additionally, we observed a gradual increase in photoluminescence in the 700–1000 cm^−1^ spectral range.

From Table 1, it can be observed that in the HDPE/TiN samples, there was a decrease in the Raman peaks (1054, 1123, 1290, and 1412 cm^−1^), which refer to skeletal vibrations (C-O-C stretching) and the methylated parts of the molecule (CH_3_ deformation). This fact, in combination with the increase in Raman C-H peaks at (2843, 2851, 2885, and 2920 cm^−1^) could be explained by the replacement of the CH_3_ bond with a CH_2_ or CH bond. Considering that the dissociation energies of CH_2_ and CH are larger than those of CH_3,_ the aforementioned differences in Raman peak intensities could explain the observed improvement in the mechanical properties of the sample [104]. In particular, the dissociation energies of the bonds within a polymer have a profound effect on its mechanical properties. The bond dissociation energy directly influences various mechanical properties, such as tensile strength, toughness, flexibility, and thermal stability.

The addition of TiN nanoparticles can induce various chemical and physical changes within the polymer matrix. One plausible explanation for the observed reduction in CH₃ bond concentration and the increase in CH₂ bond concentration is that TiN nanoparticles catalyze the dehydrogenation of CH₃ groups, effectively converting them into CH₂ groups. This dehydrogenation process involves the removal of a hydrogen atom (or hydrogen atoms) from the CH₃ groups. The fate of the detached hydrogen atoms (H) can be understood through several possible mechanisms: (i) The hydrogen atoms detached from the CH₃ groups can react with oxygen present in the polymer matrix or the surrounding environment to form hydroxyl (–OH) groups. This is particularly likely because the polymer matrix contains oxygen and is exposed to air. (ii) If two hydrogen atoms are detached from the polymer, they can combine with an oxygen atom to form water molecules. This reaction is feasible if sufficient oxygen is available and (iii) the detached hydrogen atoms also migrate within the polymer matrix and form hydrogen bonds with other functional groups present in the polymer. This can lead to changes in the physical properties and cross-linking density of the polymer, which could also be the reason for the improvement of the mechanical properties.

A sharp increase in the HDPE/TiN sample (8.0 wt. %) appearing between 500 and 700 cm^−1^ is a pseudo-increase and the result of two steps of the processing process. Initially, the polyonymic background subtraction increased this band. Second, the normalization process resulted in a non-realistic Raman signal increase at the edges of the Raman spectrum.

### 3.2. TGA and DSC Analysis Results

**Figure 5:**
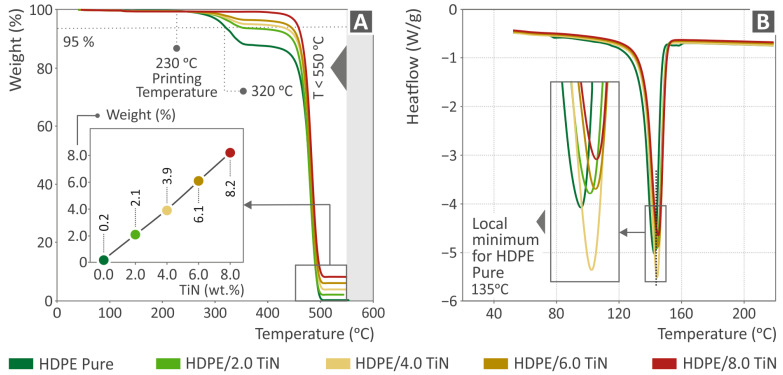
(**A**) The thermogravimetric analysis results and an inserted graph of the weight percentage as to all the HDPE/TiN filler loadings and pure HDPE. (**Β**) The differential scanning calorimetry findings of HDPE (unfilled) and all the composites (HDPE/TiN).

Figure 5 shows the thermal properties of the unfilled HDPE and the composites prepared herein (HDPE/TiN) derived from TGA and DSC analyses. Figure 5A shows the TGA results, and an inset graph of all the samples’ weight percentages in relation to their fillers is also cited, indicating that as the percentage of the additive increased, the residual weight also increased. No remarkable behavioral differences were observed between the composite samples. The inclusion of TiN slightly enhanced the thermal stability of the HDPE polymer, with the unfilled HDPE beginning to degrade at lower temperatures compared to the HDPE/TiN composites before its acute degradation at a temperature similar to that of the composites.

It should be noted that during the TGA measurements, an alteration in the chemical structure of the polymer occurred during the depolymerization (thermolysis) processes. In these processes, Polyethylene is converted into a condensable liquid product with a high concentration of straight-chain *α*-olefins [105]. The kinetics of thermal degradation and the activation energy of PEs during TGA measurements in an argon environment have been reported, and equations have been presented to assess the phenomena occurring in polymeric materials during thermal degradation [106,107,108,109]. Additionally, solid carbon residue is not formed despite an inert atmosphere, which is a common behavior of Pes, as reported in the literature [110]. The behavior of HDPE at 250–300 °C, at the beginning of HDPE degradation, is a common behavior of HDPE in TGA measurements, as presented in the literature [111,112]. In the nanocomposites, this behavior disappeared in the presence of TiN particles. This was because the liquefaction of HDPE was delayed by the addition of TiN [113,114]. Figure 5B shows the DSC curves, which also highlights that the local minimum for pure HDPE was found at 135 °C. Notably, the HDPE/TiN 4.0 wt. % sample presented the lowest heat flow values in relation to the rest of the composites.

### 3.3. Viscosity and MFR Analyses

**Figure 6:**
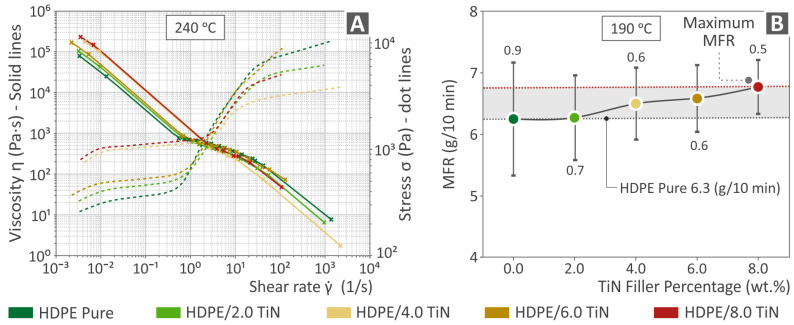
(**A**) Stress and viscosity shear rate graphs (at 240 °C) of HDPE (unfilled) and HDPE/TiN 2.0–8.0 wt. %. (**B**) MFR versus TiN filler percentage at 190 °C of HDPE (unfilled) HDPE/TiN 2.0–8.0 wt. %.

Figure 6A shows viscosity and stress versus shear rate graphs at 240 °C for the unfilled HDPE and composites (HDPE/TiN) with 2.0–8.0 wt. % TiN. As the viscosity decreased, stress increased. Figure 6B illustrates the melt flow rate (MFR) as a function of the filler percentage at 190 °C for the pure HDPE and HDPE/TiN composites with 2.0–8.0 wt. % TiN. The data indicated that a higher filler percentage resulted in an increased MFR, although the increase was within a narrow range.

The maximum value was detected for HDPE/TiN 8.0 wt. %; still, the overall differences are not significant. Similar to the viscosity and stress, an increase was found mainly at the lower shear rate values. As expected, the introduction of TiN affected the rheological properties of HDPE. However, the extent of this effect does not warrant further investigation or analysis.

### 3.4. Monitoring of Filament

**Figure 7:**
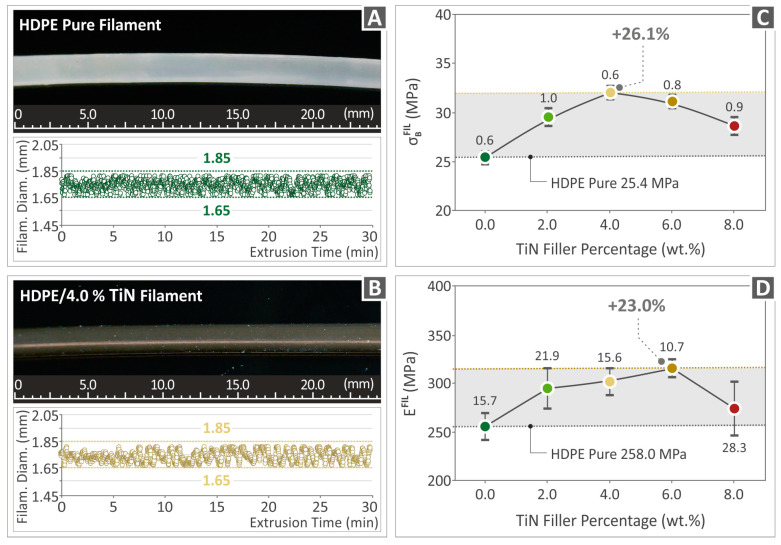
Filament assessment. (**A**,**B**) Pictures indicating the quality and the monitoring of the diameters of the pure HDPE and HDPE/TiN 4.0 wt. %. (**C**,**D**) Mechanical properties of the filaments for the tensile strength and modulus of elasticity of the unfilled HDPE and HDPE/TiN 2.0–8.0 wt. %.

In Figure 7A,B, images from the microscope captured during the inspection of filaments made of pure HDPE and HDPE/TiN 4.0 wt. %, along with the respective filaments’ diameter monitoring, are presented. The images of the filaments indicated smooth surfaces of great quality, while their diameters were kept within 1.65 mm and 1.85 mm, a range acceptable for proper 3D-P. Figure 7C,D presents the results derived from the tensile strength and modulus of elasticity tests of the unfilled HDPE and HDPE/TiN composite filaments, respectively. The highest tensile strength was detected for HDPE/TiN 4.0 wt. % by being 26.1% higher than that of the unfilled HDPE. The highest modulus of elasticity in the tensile test was reported for HDPE/TiN 6.0 wt. % by being 23.0% above that of the pure HDPE.

### 3.5. Mechanical Tests Results

**Figure 8:**
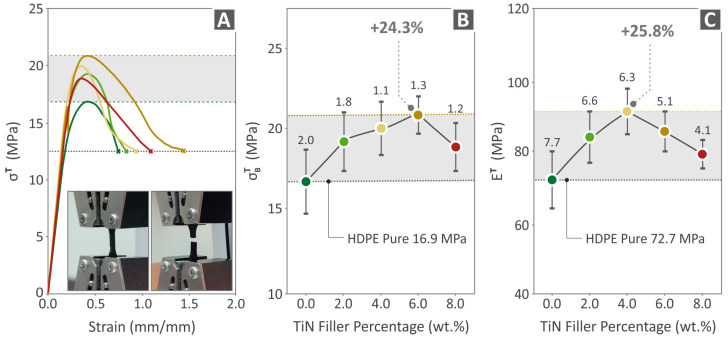
Results regarding the (**A**) curves illustrating tensile stress vs. strain, as derived from the experimental process, (**B**) tensile strength, and (**C**) the tensile modulus of elasticity of HDPE (unfilled) and HDPE/TiN 2.0–8.0 wt. %.

Figure 8 presents the experimental results of the tensile testing of the unfilled HDPE and HDPE/TiN 2.0–8.0 wt. % specimens (MEX 3D-P), namely tensile-stress-to-stain graphs (Figure 8A), tensile strength (Figure 8B), and the modulus of elasticity (Figure 8C). The highest tensile strength was observed for HDPE/TiN 6.0 wt. % (24.3% above that of pure HDPE), while the greatest value of tensile modulus of elasticity was reported at HDPE/TiN 4.0 wt. % (25.8% higher than that of pure HDPE).

**Figure 9:**
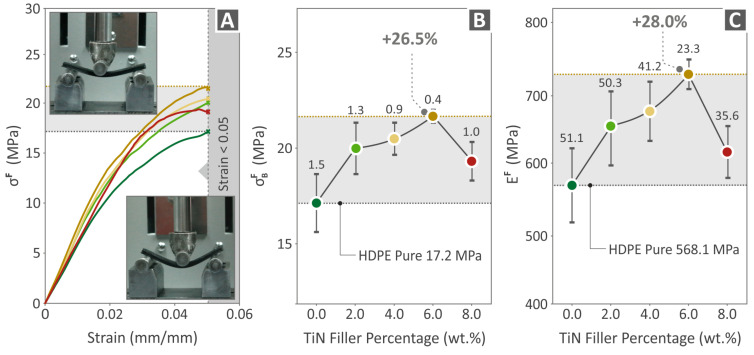
Results regarding the (**A**) curves illustrating flexural stress vs. strain, as derived from the experimental process, (**B**) flexural strength, and (**C**) the flexural modulus of elasticity of HDPE (unfilled) and HDPE/TiN 2.0 wt. %, 4.0 wt. %, 6.0 wt. %, and 8.0 wt. %.

Figure 9 presents the experimental results of the flexural testing of the unfilled HDPE and HDPE/TiN 2.0–8.0 wt. % specimens (MEX 3D-P), namely flexural-stress-to-stain graphs (Figure 9A), flexural strength (Figure 9B), and the flexural modulus of elasticity (Figure 9C). The highest flexural strength was observed for HDPE/TiN 6.0 wt. % (26.5% above that of the pure HDPE), while the most increased flexural modulus of elasticity was reported at HDPE/TiN 6.0 wt. % (28.0% above that of the pure HDPE).

**Figure 10:**
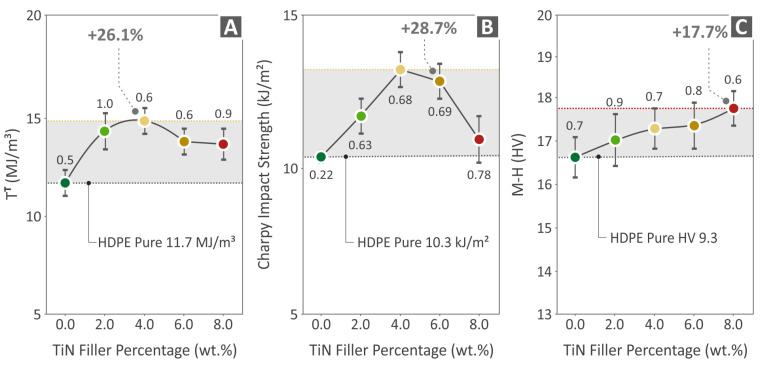
Results of the (**A**) tensile toughness, (**B**) impact strength (following the Charpy protocol), and (**C**) microhardness (Vickers) of HDPE (unfilled) and HDPE/TiN 2.0–8.0 wt. %.

Figure 10 shows the results from the unfilled HDPE and HDPE/TiN 2.0–8.0 wt. % specimens’ tensile toughness (Figure 10A), Charpy impact strength (Figure 10B), and (Vickers) microhardness (Figure 10C). The highest tensile toughness was observed for HDPE/TiN 4.0 wt. % (improved by 26.1% compared to that of the unfilled HDPE), the highest value of Charpy impact strength was found for HDPE/TiN 6.0 wt. % (28.7% above that of the pure HDPE), and last but not least, the microhardness of HDPE/TiN 8.0 wt. % was the greatest among all (17.7% above that of the pure HDPE).

### 3.6. Micro-CT of the Specimens

**Figure 11:**
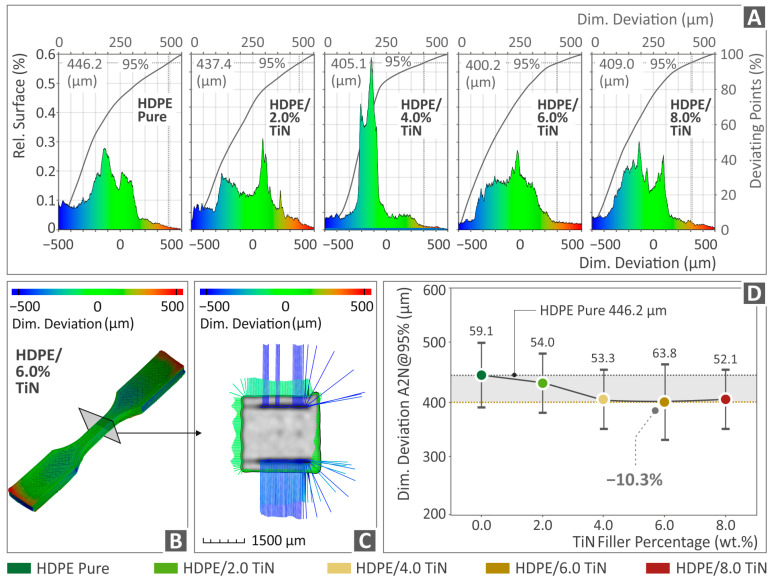
(**A**) Dimensional deviation graphs of the pure HDPE and HDPE/TiN 2.0–8.0 wt. %. (**B**,**C**) The deviation of the HDPE/TiN 6.0 wt. % tensile specimen on a specific section near the middle of the part, presented through color-coded mapping. (**D**) The A2N dimensional deviation at 95% of HDPE (unfilled) and HDPE/TiN 2.0–8.0 wt. %.

**Figure 12:**
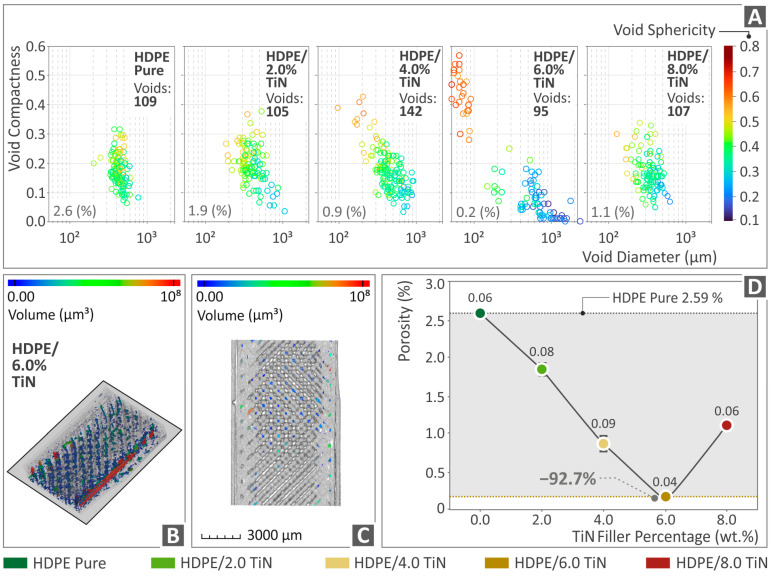
(**A**) Void graphs of pure HDPE and HDPE/TiN 2wt. %, 4wt. %, 6wt. %, and 8wt. %. (**B**,**C**) Visual representation by color-coding mapping of porosity of HDPE/TiN 6.0 wt. % specimens, and (**D**) porosity percentage of HDPE (unfilled) and HDPE/TiN 2.0–8.0 wt. %.

Figure 11 and Figure 12 show the results derived from the μ-CT scanning of HDPE (unfilled) and HDPE/TiN 2.0–8.0 wt. %, regarding the dimensional accuracy (deviation from the nominal geometry) and porosity of the respective parts. Statistical analysis was used to present the dimensional deviation of the 3D-P HDPE pure and all the HDPE/TiN composite specimens (Figure 11A). Moreover, the HDPE/TiN 6.0 wt. % sample presented using color coding presents the comparison of its geometry deviations from the nominal ones defined in the CAD file (Figure 11B,C). Figure 11D shows the A2N dimensional deviation at 95% (5% of the most extreme values were discarded) for the pure HDPE and HDPE/TiN samples. HDPE/TiN 6.0 wt. % was the composite that presented the lowest value below that of the pure HDPE (10.3% lower). The addition of TiN to all the composites provided HDPE with an improvement in dimensional accuracy compared to that of the pure HDPE.

The 3D-P specimens of HDPE (unfilled) and all the composites (HDPE/TiN) were examined in terms of their porosities, and the results are shown in Figure 12. The graphs in Figure 12A show the void distribution and shape (sphericity). Figure 12B,C provides visual representations of the porosity distribution and size (volume), particularly for the HDPE/TiN 6.0 wt. % composite for a specific cross-section. Finally, Figure 12D shows the porosity percentage values for both the pure HDPE and HDPE/TiN composite samples. The findings revealed that the incorporation of TiN into HDPE had a notable impact on porosity. In particular, for HDPE/TiN 6.0 wt. %, the decrease in porosity in relation to that of the pure HDPE was spectacular, as it reduced by 92.7%. However, it is worth noting that after increasing the filler percentage, porosity showed an upward trend.

### 3.7. Morphological Characteristics of the Samples, Evaluated with SEM

**Figure 13:**
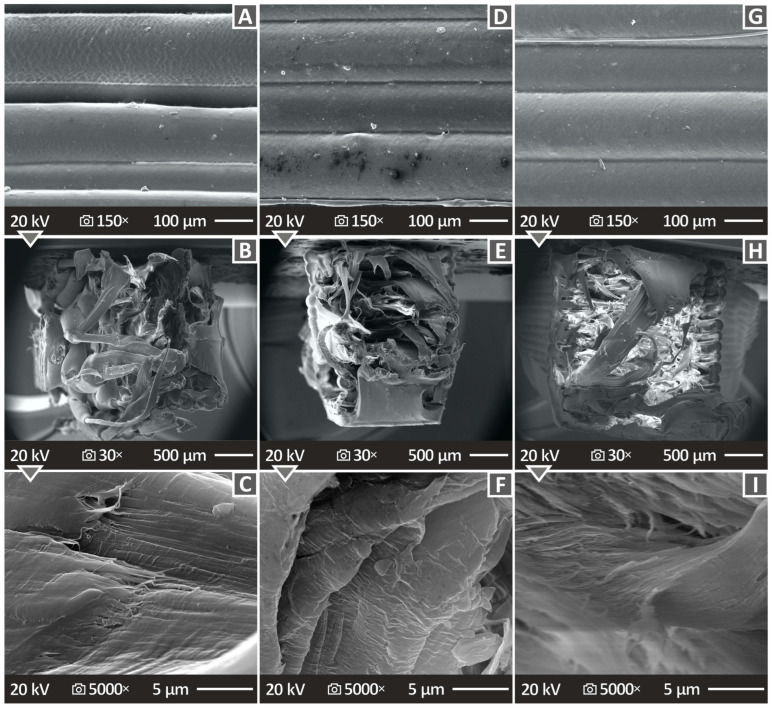
(**A**–**C**) HDPE/TiN 2.0 wt. % SEM pictures of the lateral area magnified 150×, fracture area magnified at 30× and 5000×. (**D**–**F**) HDPE/TiN 4.0 wt. % SEM illustrations of the lateral area at 150× magnification, fracture area at 30× and 5000× magnifications, and (**G**–**I**) HDPE/TiN 8.0 wt. % SEM pictures of the lateral area magnified at 150× and fracture area magnified at 30× and 5000×.

**Figure 14:**
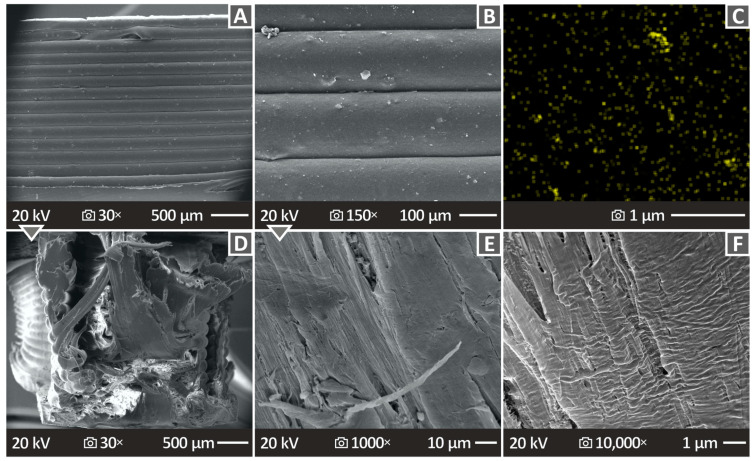
(**A,B**) Lateral area illustration of HDPE/TiN 6.0 wt. % magnified at 30× and 150×, correspondingly. (**C**) EDS mapping image for the TiN element of a selected region. (**D**–**F**) Fracture section illustrations of HDPE/TiN 6.0 wt. % magnified at 30× and 1000× and 10,000×, respectively.

Figure 13 and Figure 14 show the SEM images of the fracture and side planes of the manufactured 3D-P parts. Figure 13A,D,G illustrates the side surfaces of the HDPE/TiN 2.0 wt. %, HDPE/TiN 4.0 wt. %, and HDPE/TiN 8.0 wt. % samples, magnified at 150×. No defects or voids were observed, and the layers exhibited a uniform distribution. Figure 13B,E,H shows illustrations of the fracture surfaces at 30× magnification of the composites with 2.0, 4.0, and 8.0 wt. % TiN contents. The same samples at 5000× magnification are shown in Figure 13C,F,I. Irregular fibers were detected at a magnification of 30×. At 5000× magnification, the HDPE/TiN 2.0 wt. % showed a great surface quality, mostly smooth, and the HDPE/TiN 4.0 wt. % and HDPE/TiN 8.0 wt. % presented more uneven surfaces, while in all of them, a few pores and voids made their appearance.

Figure 14 not only includes SEM images (Figure 14A,B,D–F), but also an EDS image (Figure 14C), all regarding the specimen samples taken from HDPE/TiN 6.0 wt. %. Figure 14A,B shows the lateral area SEM images at 30× and 150× magnifications, respectively. From the perspective of the side surface point of view, it can be concluded that the fusion is well distributed without pores, voids, or defects. As already mentioned in Figure 14C, there is an EDS mapping image of the derived titanium nitride (TiN) element sample. The elemental distribution cannot be characterized as being well distributed, which probably indicates the presence of particle clustering in the nanocomposite with this filler percentage. Figure 14D–F shows SEM images of the fracture section at magnifications of 30×, 1000×, and 10,000×, respectively. The surfaces are mostly smooth. Minor defects were observed, particularly at a magnification of 30×.

## 4. Discussion

The incorporation of TiN filler enhanced the mechanical properties of the samples. Specifically, the HDPE/TiN 4.0 wt. % composite exhibited an improved tensile modulus of elasticity and tensile toughness, while the HDPE/TiN 6.0 wt. % composite demonstrated an increased tensile strength, flexural strength, modulus of elasticity, and Charpy impact strength. Additionally, the HDPE/TiN 8.0 wt. % composite showed the highest value compared to the pure HDPE in terms of microhardness. However, the deterioration in most of the mechanical properties of the HDPE/TiN 8.0 wt. % composites suggests that the additive reached a saturation point within the matrix. Therefore, further increases in the TiN content were not expected to result in better outcomes. The precise saturation threshold remains undefined as it was not investigated within the confines of the current study. 

In this study, the tensile strength of the filaments was tested. The results followed a pattern similar to that of the 3D-printed samples. For the filament, the highest improvement was observed for the 4 wt. %-loaded composite, while in the 3D-printed samples, the 6 wt. %-loaded composite reported the highest tensile strength values. The 6 wt. %-loaded filament showed similar strength to the 4 wt. % one (a small decrease in the tensile strength value). At higher loadings in both the filament and 3D-printed parts, the tensile strength decreased. The overall improvements in the tensile strength and modulus were also similar between the filament and the 3D-printed parts (~25%). The filament was not tested according to a standard, and, to the best of our knowledge, there is no relevant standard for these tests. Therefore, the acquired results cannot be correlated with the respective results for the 3D-printed parts, which follow a standard, and the 3D-printed structure considerably affected the mechanical performance of the samples. Nevertheless, the findings are similar, as mentioned, showing the consistency and reliability of the reported findings of this research. In terms of absolute values, the tensile strength and modulus were considerably higher than those of the 3D-printed samples. Such a difference is expected because of the differences between the filament and 3D-printed samples mentioned above (the solid structure of the filaments and the tests not following a standard).

The limitations of this study are related mainly to the overall limitations of the 3D-printed parts [115,116]; these are dimensional accuracy (which was evaluated and reported within the context of the study), the lack of usefulness in mass production, the high cost compared to that of injection-molded parts, and the lower strength than that of bulk parts. This last parameter triggered this research in an effort to improve the mechanical performance of parts made with HDPE via 3D printing. To improve the mechanical performance, the introduction of TiN nanoparticles increases the cost of the parts made with these nanocomposites. However, the cost increase is related only to the cost of the procurement of the nanoparticles, which cannot be considered significant because of the low content of nanoparticles in the nanocomposites.

Considering the μ-CT scanning results, the dimensional deviation was slightly improved in the composite with 6.0 wt. % TiN by 10.3% in relation to that of the unfilled HDPE, while porosity revealed a spectacular improvement by the same composite, being 92.7% lower than the porosity of the pure HDPE. The reduction in porosity contributes to an improvement in the mechanical performance of the 3D-printed parts. This is owing to the increase in the section of the 3D-printed parts and the more robust structure of the samples [117,118,119]. The addition of TiN reduced the porosity of the samples by up to 6 wt. % content. Beyond this TiN percentage in the samples, the increased porosity contributed to the worse mechanical properties of the 3D-printed parts. This increase in porosity with increasing TiN content in the samples beyond 6 wt. % can be attributed to the effect of TiN in the rheological properties of the composites, which is presented in Figure 6. The viscosity increased in the 8 wt. % samples, and so did the stress. This alteration in the rheological behavior of the composites leads to changes in the fusion between layers and increased porosity in the 3D-printed structure.

**Figure 15:**
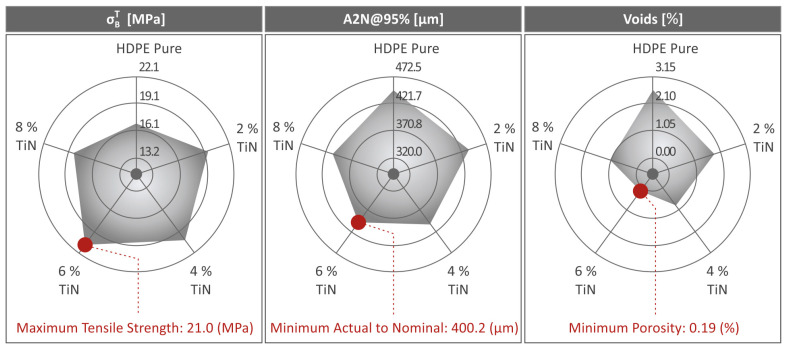
Summarization of tensile strength, A2N at 95%, and void results in three respective spider-shaped graphs regarding the pure HDPE and HDPE/TiN 2wt. %, 4wt. %, 6wt.%, and 8wt. %.

Figure 15 shows three different spider-shaped graphs that summarize the pure HDPE and all the HDPE/TiN composite values regarding tensile strength, the actual-to-nominal dimensional deviation at 95%, and the voids. The most remarkable values were also highlighted to identify the HDPE/TiN composites to which they belonged. Overall, the addition of TiN nanoparticles improved not only the mechanical performance of the HDPE polymer, but also the quality aspects of the 3D-P parts, such as dimensional accuracy and porosity. However, their rheological properties were not significantly altered. This resulted in good layer fusion and uniform layer shaping, even for the higher-loaded composites. A more notable change in the rheological properties would have affected the 3D-P quality, suggesting an adjustment of the 3D-P parameters for each composite. This seems unnecessary.

High-magnification images captured using a scanning electron microscope (SEM) were used to scrutinize the formation of agglomerations in the 3D-printed samples. The primary objective of these images was to pinpoint potential agglomerations within the materials and appraise the dispersion quality of the nanofiller. The fracture surfaces of the samples with the highest filler concentrations were examined using SEM analysis, which revealed unavoidable agglomerations. These agglomerations have a significant impact on the mechanical properties, as discussed in this study. In contrast, the samples with lower filler concentrations displayed no agglomerations. This finding suggests that the nanofiller created a nanoscale network within the polymer matrix, although some fillers may not have achieved complete dispersion. Energy-dispersive X-ray spectroscopy (EDS) analysis conducted across different fracture surface regions confirmed these observations. Moreover, mechanical testing indicated that the variability in the results was within acceptable limits, suggesting a consistent composition among all the nanocomposite samples studied. These findings suggest that the process followed for the preparation of the nanocomposites, which, at the same time, is compatible with the MEX 3D printing process as already presented, managed to achieve a good dispersion of the filler in the matrix.

To the best of our knowledge, no prior studies have investigated the performance of 3D-P HDPE/TiN composites with varying filler percentages, specifically MEX 3D-P. However, investigations have been conducted in which the same TiN filler used in this study was employed on different matrix materials. For instance, a part of one [87] investigation examined the reinforcing properties of TiN on polyamide 12 (PA12). The results showed that the composites showed a significant improvement in their mechanical performance compared to that of pure PA12, which also occurred in the HDPE. In this [88] study, the efficiency of TiN filler in the performance of pure polypropylene (PP) was investigated, and it was found that there was an enhancement provided by the filler. Polylactic Acid (PLA) has also been combined with TiN, and its mechanical reinforcement capability has been investigated, indicating, for one more time, promising results with improved mechanical properties compared with those of pure PLA [89]. To examine HDPE combined with different fillers [120], TiO_2_ was employed and investigated for its reinforcing properties, revealing greatly enhanced behavior, mostly in the case of the low filler percentages. How the TiN nanoparticles have an impact on the mechanical response of different polymeric matrices is presented in Table 2. The literature includes studies in which nanocomposites in MEX 3D-P were prepared using a method similar to that used in the current study. Differences can be observed, which are expected and justify the need for separate studies for each matrix as the interactions with the filler differ. A similar response was observed for the PC [90] and PA12 [87] polymers, with a difference in the impact strength, in which PA12 showed the second-greatest improvement among the polymers tested. On the other hand, ABS [71] showed a smaller enhancement than the remaining polymers. In particular, the impact strength decreased with the addition of TiN particles. However, the improvement in microhardness was the best among the polymers. An increase in microhardness is expected owing to the high hardness of TiN material. The PP [88] and PLA [89] polymers showed an overall higher improvement in their mechanical performance with the addition of TiN particles. The PLA [89] polymer showed the highest increase in impact strength, with an impressive 91.4% improvement over that of the pure matrix material. Overall, the highest improvement in all the polymeric matrices was achieved with filler loadings ranging from 2.0 to 6.0 wt. %. The HDPE studied herein achieved the biggest improvement overall, with a 6.0 wt. % filler percentage, which is not high and something typical for nanoparticles; still, it is three times more than the amount the PP [88] polymer required, which also achieved higher improvement in its properties. This is also a metric for the different effects of fillers in matrix materials.

## 5. Conclusions

In the present study, four different nanocomposites of HDPE/TiN were examined for their performance in relation to pure HDPE with TiN contents of 2.0, 4.0, 6.0, and 8.0 wt. %. Initially, they were created in the form of mixtures, which were turned into their respective filaments, and then into specimens, which were acceptable for participating in the upcoming mechanical tests. The prepared samples were subjected to comprehensive analysis of their thermal, rheological, mechanical, structural, and morphological properties. Mechanical testing included evaluations of the tensile, flexural, and Charpy impact strengths, as well as microhardness assessments. SEM was also performed on the fabricated specimens, with the aim of showing the fracture and side surface characteristics. The μ-CT scanning results were evaluated by examining the porosity and the dimensional deviation of the samples. The porosity reduction in the HDPE/TiN 6.0 wt. % composite was spectacular, with a 92.7% decrease in relation to that of the unfilled HDPE. Overall, the mechanical properties were enhanced by the addition of TiN to the HDPE matrix material. HDPE/TiN 6.0 wt. % was the composite with an overall better mechanical performance among the mixtures tested. The addition of TiN improved aspects of the 3D-printed samples, such the dimensional accuracy, porosity, and the thermal stability of the composites. As expected, because of the hard ceramic TiN particles, the improvement was bigger for the composite with a higher filler percentage. Additional filler percentages could be investigated in future studies to assess their performance (for example, for wear resistance or defense applications, owing to the nature of the filler), and other aspects should be investigated as well related to the industrialization of the process.

## Figures and Tables

**Table 1:** Significant differences in Raman peak intensities were observed between the HDPE pure and HDPE/TiN samples with the following assignments.

Wavenumber (cm^−1^)	Raman Spectrum Changes
700–1000	Gradual increase in photoluminescence
1054	Gradual decrease C-O-C stretching [99]
1123	Gradual decrease in C-O-C stretching [100]
1290	Gradual decrease in C-O-C stretching [99]
1412	Gradual decrease in CH_3_ deformation [99]
2843	Gradual increase CH_2_ symmetric stretching [102]
2851	Gradual increase in CH_2_ symmetric stretching [102]
2885	Gradual increase in CH_2_—CH symmetric stretching [102,103]
2920	Gradual increase in CH_2_ asymmetric stretching [102]

**Table 2:** Comparison of the reinforcing effect of TiN in different polymeric matrices for composites developed for the MEX 3D printing method.

Increase	HDPE (Current Study)	ABS [71]	PA12 [87]	PP [88]	PLA [89]	PC [90]
Tensile strength	24.3	18.1	45.5	41.5	43.4	35.5
Flexural strength	26.5	36.9	23.0	33.7	51.5	13.6
Impact strength	28.7	decrease	64.3	18.0	91.4	16.2
microhardness	17.7	62.7	22.2	44.8	27.3	20.2
Optimum filler loading (wt. %)	6.0	6.0	2.0	2.0	4.0	3.0

## Data Availability

The raw/processed data required to reproduce these findings cannot be shared because of technical and time limitations.

## Supplementary Materials

The following supporting information can be downloaded from: https://www.mdpi.com/article/10.3390/polym16121702/s1, Table S1: Significant Raman peaks were observed, along with their related assignments, from pure HDPE.
