# Supplementary material for: Optimization Course of Titanium Nitride Nanofiller Loading in High-Density Polyethylene: Interpretation of Reinforcement Effects and Performance in Material Extrusion 3D Printing
Source: Polymers (Basel). 2024 Jun 14;16(12):1702. doi: 10.3390/polym16121702 (PMC11207989; doi:10.3390/polym16121702)
Supplement: Supplementary file 1 [file polymers-16-01702-s001.zip › polymers-3052225-supplementary.pdf]

polymers

MDPI

Supplementary material for article

Optimization course of titanium nitride nanofiller loading in high-density polyethylene: interpretation of reinforcement effects and performance in material extrusion 3D printing

Markos Petousis [a], Dimitris Sagris [b], Vassilis Papadakis [c, d], Amalia Moutsopoulou [a], Apostolos Argyros [e, f], Constantine David [b], John Valsamos [a], Mariza Spiridaki [a], Nikolaos Michailidis [e, f] and Nectarios Vidakis [a,*]

[a] Department of Mechanical Engineering, Hellenic Mediterranean University, Heraklion 71410, Greece, markospetousis@hmu.gr; amalia@hmu.gr, valsamos@hmu.gr; mspyridaki@hmu.gr; vidakis@hmu.gr;
[b] Department of Mechanical Engineering, International Hellenic University, Serres Campus, 62124, Greece, dsagris@ihu.gr; david@ihu.gr;
[c] Institute of Electronic Structure and Laser of the Foundation for Research and Technology-Hellas (IESL-FORTH) – Hellas, N. Plastira 100m 70013 Heraklion, Greece
[d] Department of Industrial Design and Production Engineering, University of West Attica, 122 43 Athens, Greece, v.papadakis@uniwa.gr
[e] Physical Metallurgy Laboratory, Mechanical Engineering Department, School of Engineering, Aristotle University of Thessaloniki, 54124 Thessaloniki, Greece, aargyros@auth.gr; nmichail@auth.gr;
[f] Centre for Research & Development of Advanced Materials (CERDAM), Centre for Interdisciplinary Research and Innovation, Balkan Centre, Building B', 10th km Thessaloniki-Thermi road, 57001, Thessaloniki, Greece
* Correspondence: author: e-mail: vidakis@hmu.gr (Nectarios Vidakis), Tel.: +302810379227

Raman results of HDPE/ TiN composites

Table S1. Significant Raman peaks along with their related assignments from HDPE pure.

Wavenumber (cm^{-1})	Intensity	Raman peak assignment
1064	Medium	C-O-C stretching [1]
1131	Medium	C-O-C stretching [2]
1297	Medium	C-O-C stretching [1]
1418	Small	CH_3 deformation [1]
1441	Small	CH_2 deformation [1,3]
2850	Major	CH_2 symmetric stretching [4]
2884	Major	C-H antisymmetric stretching [5]

References

1. Stuart, B.H. Temperature Studies of Polycarbonate Using Fourier Transform Raman Spectroscopy. *Polymer Bulletin* **1996**, *36*, 341–346, doi:10.1007/BF00319235.
2. Resta, V.; Quarta, G.; Lomascolo, M.; Maruccio, L.; Calcagnile, L. Raman and Photoluminescence Spectroscopy of Polycarbonate Matrices Irradiated with Different Energy 28Si+ Ions. *Vacuum* **2015**, *116*, 82–89, doi:10.1016/j.vacuum.2015.03.005.
3. Zimmerer, C.; Matulaitiene, I.; Niaura, G.; Reuter, U.; Janke, A.; Boldt, R.; Sablinskas, V.; Steiner, G. Nondestructive Characterization of the Polycarbonate - Octadecylamine Interface by Surface Enhanced Raman Spectroscopy. *Polym Test* **2019**, *73*, 152–158, doi:10.1016/j.polymertesting.2018.11.023.

4. Makarem, M.; Lee, C.M.; Kafle, K.; Huang, S.; Chae, I.; Yang, H.; Kubicki, J.D.; Kim, S.H. Probing Cellulose Structures with Vibrational Spectroscopy. *Cellulose* **2019**, *26*, 35–79, doi:10.1007/s10570-018-2199-z.
5. Liu, X.; Zou, Y.; Li, W.; Cao, G.; Chen, W. Kinetics of Thermo-Oxidative and Thermal Degradation of Poly(d,l-Lactide) (PDLLA) at Processing Temperature. *Polym Degrad Stab* **2006**, *91*, 3259–3265, doi:10.1016/j.polymdegradstab.2006.07.004.

Disclaimer/Publisher's Note: The statements, opinions and data contained in all publications are solely those of the individual author(s) and contributor(s) and not of MDPI and/or the editor(s). MDPI and/or the editor(s) disclaim responsibility for any injury to people or property resulting from any ideas, methods, instructions or products referred to in the content.
